# Preparation of Electrochemical Supercapacitor Based on Polypyrrole/Gum Arabic Composites

**DOI:** 10.3390/polym14020242

**Published:** 2022-01-07

**Authors:** Rizwan Ullah, Nadia Khan, Rozina Khattak, Mehtab Khan, Muhammad Sufaid Khan, Omar M. Ali

**Affiliations:** 1 National Center of Excellence in Physical Chemistry, University of Peshawar, Peshawar 25120, Pakistan; Khannadia@uop.edu.pk (N.K.); mehtabk324@uop.edu.pk (M.K.); 2 Department of Chemistry, Shaheed Benazir Bhutto Women University, Peshawar 25000, Pakistan; 3 Department of Chemistry, University of Malakand, Chakdara 18800, Pakistan; sufaidkhan1984@uom.edu.pk; 4 Department of Chemistry, Turabah University College, Turabah Branch, Taif University, P.O. Box 11099, Taif 21944, Saudi Arabia; om.ali@tu.edu.sa

**Keywords:** polypyrrole, gum arabic, supercapacitors, EIS, GCD

## Abstract

The current research focused on the super capacitive behavior of organic conducting polymer, i.e., polypyrrole (PPy) and its composites with gum arabic (GA) prepared via inverse emulsion polymerization. The synthesized composites material was analyzed by different analytical techniques, such as UV-visible, FTIR, TGA, XRD, and SEM. The UV-Vis and FTIR spectroscopy clearly show the successful insertion of GA into PPy matrix. The TGA analysis shows high thermal stability for composites than pure PPy. The XRD and SEM analysis show the crystalline and amorphous structures and overall morphology of the composites is more compact and mesoporous as compared to the pure PPy. The electrochemical properties of modified solid state supercapacitors established on pure polypyrrole (PPy), polypyrrole/gum arabic (PPy/GA) based composites were investigated through cyclic voltammetry (CV), electrochemical impedance spectroscopy (EIS) and galvanostatic charge–discharge (GCD). The specific capacitance of the PPy modified gold electrode is impressive (~168 F/g). The specific capacitance of PPy/GA 1 electrode has been increased to 368 F/g with a high energy density and power density (~73 Wh/kg), and (~599 W/kg) respectively.

## 1. Introduction

In the modern era, an integral part of human life is smart technology. Accordingly, advanced technologies are always searching for smart and well-fabricated materials to satisfy the growing demand [1,2,3]. The development of novel materials with improved electrochemical performance is required to address the critical issue of pollution. There is a growing need for sustainable and renewable energy storage solutions in hybrid automobiles and portable electronic devices [4], necessitating the development of innovative materials with better electrochemical capabilities, such as electrochemical capacitors or supercapacitors [5]. A supercapacitor is a type of energy storage system that combines both battery and conventional capacitor properties [6,7]. Electrochemical capacitors, or supercapacitors, have been extensively used in high-power energy storage materials. As such, supercapacitors are one of the most promising candidates among the various systems that lead the state-of-the-art electrical energy storage systems due to their environmental friendliness, sustainable cycle stability, low cost [8], excellent cycling life [9], high power density, and fast charging/discharging rate [5,10]. Supercapacitors are classified as electrochemical double-layer capacitors (EDLCs) or pseudosupercapacitors based on their charge storage mechanism [11,12]. The electrostatic separation of ionic and electronic charges at the electrode and electrolyte interfaces provides energy storage in EDLCs, and the efficiency of such devices is dictated by the surface area involved in the charge accumulation process between the electrodes and electrolyte [13]. However, a faradaic (redox) reaction happens at the electrode surfaces in the pseudocapacitor process, resulting in energy storage effects. The behavior of pseudocapacitors is determined by the electrode material, which exhibits electrochemical signatures, and the charge storage is mostly determined by the applied voltage [14,15]. Conducting polymers (CPs) are the best choice for these two types of electrochemical devices because they possess both ionic and electronic conductivities. Doping various ionic or non-ionic materials or fillers into CPs can further increase their conductivities [16].

Among the various CPs, PPy is extensively studied because of its ease of preparation, stabilized oxide form, good oxidation and reduction properties [17], and the capability to provide high conductivity [18], commercially easily available monomers [19], as well as optical and good electrical properties [20]. PPy consists of alternate single and double-bonded macromolecular chain structures. PPy excellent performance is due to its structure, but it also has some drawbacks, such as lower capacitance and poor cycling stability, which limit its use in high-performance supercapacitors [21]. It is well known that biodegradable polymers are preferable to non-biodegradable polymers. Some of the biodegradable polymers that are used as supercapacitorsare chitosan (CS) [22], PVA [23], and glycerol [24]. On the other hand, gum arabic (GA) in the composite form can be used to alleviate the pure PPy problem. The insertion of GA into the PPy matrix can be a promising choice due to its high contact area, chemical stability, thermal stability, and mechanical stability, as well as its high energy storage capabilities at the electrode/electrolyte interface.

In this research work, an electrode for a supercapacitor based on PPy/GA composites was fabricated by inverse emulsion polymerization. The electrochemical characteristics, cyclic voltammetry (CV), electrochemical impedance spectroscopy (EIS), and galvanometric charging–discharging (GCD) properties of the fabricated PPy/GA composites based supercapacitor devices were investigated. The synthesized materials could be promising electrode materials for high-performance supercapacitor applications, which have not been previously reported.

## 2. Materials and Methods

### 2.1. Materials

All the chemicals were of Analytical grade. Pyrrole monomer (Fluka Chemie AG, Buchs, Switzerland) was distilled twice before use. Toluene, 2-propanol, benzoyl peroxide (BPO) (≥ 98% Sigma-Aldrich Inc., ST. Louis, MO, USA), N-methyl-2-pyrrolidone (NMP) (RCI Labscan limited Pathumwan, Bangkok, Thailand), dodecylbenzene sulphonic acid (DBSA) and acetone > 98%, Sigma-Aldrich Inc., ST. Louis, MO, USA),were used as received. Double-distilled water was used for solution preparation and for washing glassware.

### 2.2. Synthesis of Polypyrrole (PPy)

**Figure 1:**
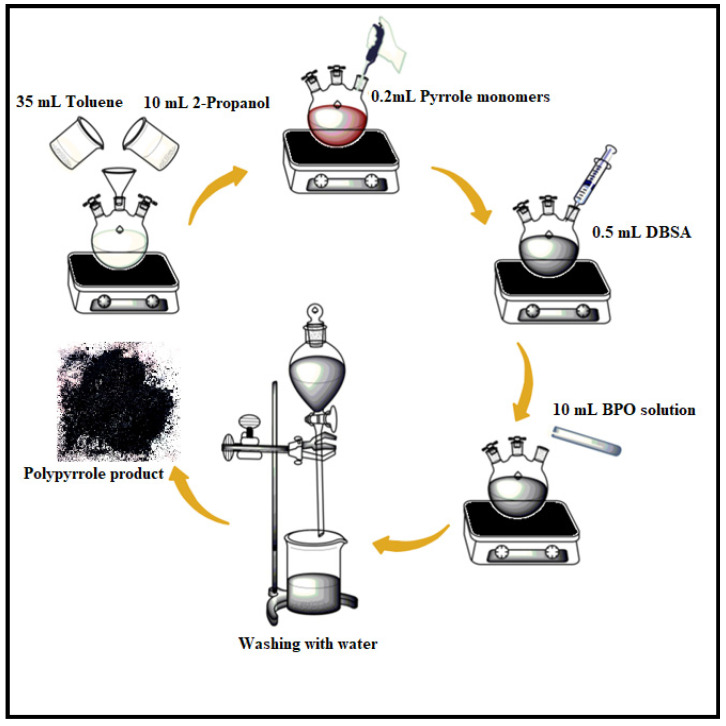
Synthesis route followed for polypyrrole.

PPy was synthesized by using a pyrrole monomer via inverse emulsion polymerization. The procedure was carried out in a three-necked round bottom flask holding 35 mL toluene and 10 mL 2-propanol that was stirred for 15 min. After that, 200 μL of pyrrole was added and stirred for another 15 min, followed by drop wise addition of 0.5 mL of DBSA and 0.303 g of benzoyl peroxide (dissolved in 5 mL of water) to the reaction mixture. To get the precipitate, the mixture was vigorously stirred for 24 h. The precipitate was washed three times with distilled water and 50 mL of acetone to separate the pure product that was dried in an oven at 50 °C for 24 h. The representative procedure of the synthesis of polypyrrole has been sketched in Figure 1.

### 2.3. Synthesis of Polypyrrole/Gum Arabic (PPy/GA) Composites

**Scheme 1:**
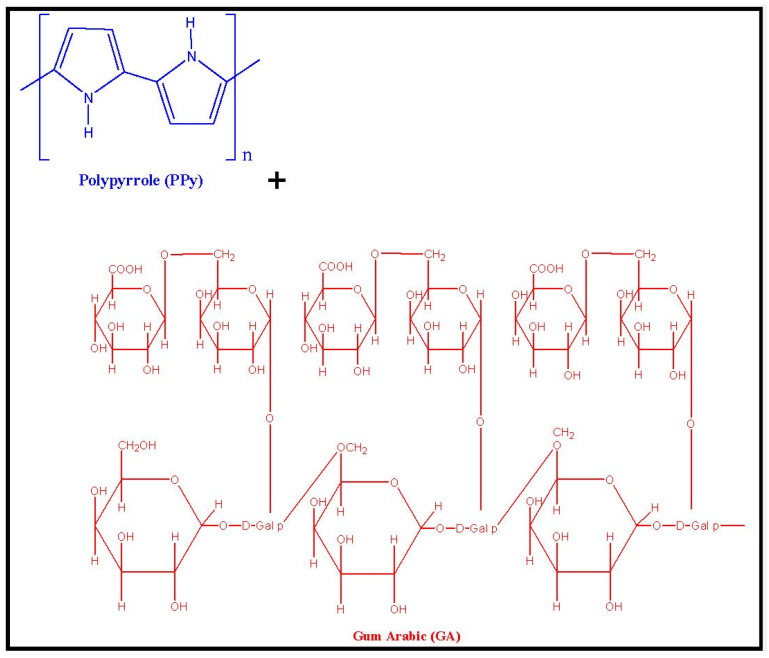
Structural representation of reactants, i.e., polypyrrole and gum arabic.

PPy/GA composites were synthesized by inverse emulsion polymerization using monomer pyrrole and gum arabic (GA). The procedure was the same as for polypyrrole synthesis, except in composite production, different weight percent’s of GA, such as 0.125%, 0.25%, 0.75% and 1% were added before the addition of benzoyl peroxide (BPO). To get the precipitate, the mixture was vigorously stirred for 24 h. To separate the pure product, the precipitate was washed three times with distilled water and 50 mL of acetone, then dried in an oven at 50 °C for 24 h. Table 1 shows the composition of PPy/GA composites and Scheme 1 shows the structural representation of the reactants.

### 2.4. Characterization of the Synthesized Polymer and its Composites with Gum Arabic

The UV/visible spectrophotometer (UV752PC) (Citi Scientific Supply, Ltd. Karachi, Pakistan) was employed to identify the electronic transitions from lower to higher energy levels in the UV-visible range of radiation. To detect functional groups and the interaction of PPy and PPy/GA composites, we employed Fourier-transform infrared (FTIR) spectroscopy (model 783 PerkinElmer Inc., Waltham, MA, USA). The crystallinity of the conducting polymers (CPs) was measured using X-ray diffractometry. The XRD spectra of the synthesized material were taken by using {Cu Kα radiations (λ = 1.5405 A°) JEOL JDX-3532, X-ray diffractometer, JEOL Ltd., Tokyo, Japan}. Scanning electron microscopy (SEM JSM-IT-100 JEOL Ltd., Tokyo, Japan) helped to scan the surface of the synthesized materials. The thermogravimetric analysis of the synthesized materials was carried out at TGA SDT Q600 (PerkinElmer Inc., Waltham, MA, USA). The electrochemical properties such as cyclic voltammetry (CV), electrochemical impedance spectroscopy (EIS), and galvanostatic charge–discharge (GCD) were performed on the electrochemical workstation (PGSTAT302, Metrohm AUTOLAB B.V. Ltd., KM Utrecht, Netherlands). The CV tests were performed at the scan rate of 100 mV/s in the potential window of −0.4 to 0.8 V. The GCD tests were conducted under current densities from 1 to 2.5 A/g by holding the cut-off voltage between −0.4 and 0.8 V.

### 2.5. Fabrication of Solid-State Supercapacitors

**Figure 2:**
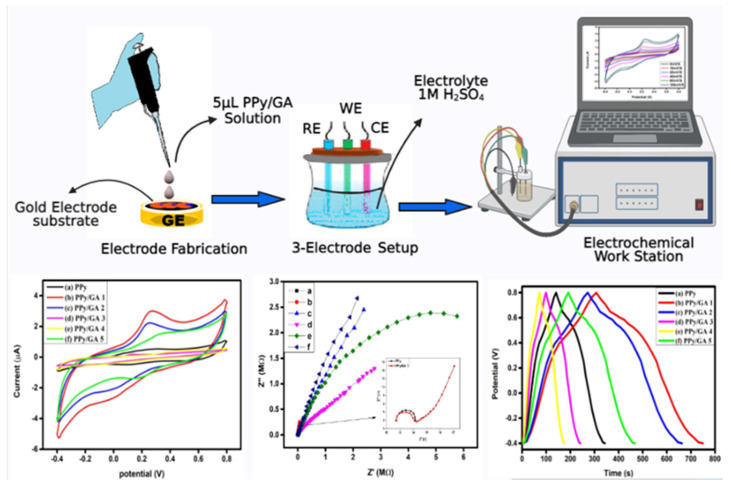
Schematic illustration of the electrode fabrication towards electrochemical properties.

The surface of the gold electrode (GE) was modified to evaluate the electrochemical activity of pure PPy and PPy/GA composites. Before using the GE, it was meticulously polished with 0.3 μm alumina (Al_2_O_3_) powder, rinsed with deionized water, then ethanol, and then sonicated for 10 min. After that, the material was ultrasonically dispersed for 5 min to prepare an effective and uniform chloroform solution of any specified composition of PPy/GA. The surface of the GE was modified using the 5 μL PPy/GA composite solution to obtain the PPy/GA/GE. The electrode was kept at room temperature for 10 min to dry completely. The surface of the electrode was activated by cycling the voltage from −0.4 to 0.8 V at a scan rate of 100 mV/s in a 1 M solution of H_2_SO_4_. The modified electrode was carefully cleaned with distilled water before and after the experiment, then reactivated using the method described above (Figure 2).

## 3. Results and Discussion

### 3.1. UV-Visible Analysis of the Synthesized PPy and PPy/GA Composites

**Figure 3:**
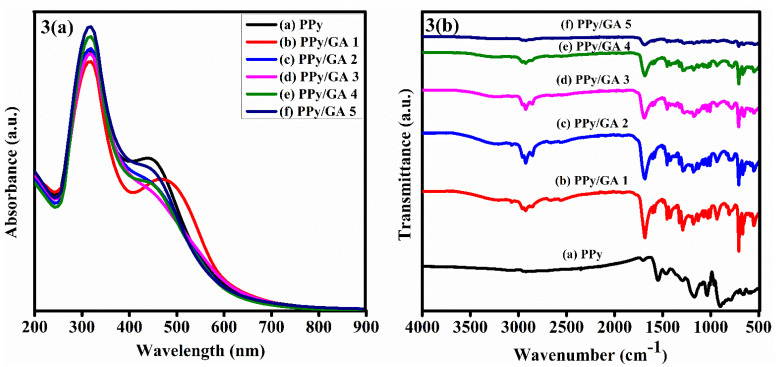
(**a**) UV-visible spectra of neat polypyrrole and its composites with gum arabic. (**b**) FTIR spectra of neat polypyrrole and its composites with gum arabic.

Figure 3a shows the UV-visible spectra of pure PPy and PPy/GA composites. Two significant absorption peaks can be seen in the spectra. At 312–319 nm and 445–480 nm, the first and second absorption peaks were found, respectively. The transition of electrons from the lowest occupied molecular orbital (LOMO) to the highest unoccupied molecular orbital (HUMO), which corresponds to the π–π* electronic transition of the aromatic ring in the polymer chain, is responsible for the first absorption band [25]. The sum of polarons and bipolarons is assigned to the second absorption band, which serves to determine that the PPy component of the composites is made up of free carriers (mainly polarons) [26], suggesting the CPs in their oxidized and conducting state [27]. The difference in peak intensities is related to the difference in composite concentration in the solvent, whereas the difference in peak position is due to the length of the polymer chain. There is a change in the absorption spectra when GA is added to the PPy matrix. The first absorption band exhibited a small rise as GA concentrations increased. Both intensity and peak shifting were detected in the second absorption peak. The absorption peak for PPy/GA 1 shifts toward a longer wavelength (red shift). The shift of peaks towards lower wavelengths was noted in the PPy/GA 2 through PPy/GA 5 composites. The absorption shift is caused by the blocking of ions or free radicals or the active site of the PPy by GA.

### 3.2. FTIR Analysis of the Synthesized PPy and PPy/GA Composites

The FTIR analysis of PPy and PPy/GA composites was performed in the range of 500 to 4000 cm^−1^ to investigate the atomic and molecular vibrations and the types of bonding states in the synthesized materials. The low-intensity peak in the PPy spectrum in the region of 2954–2851 cm^−1^ is attributable to the C–H and S=O stretching modes, which clearly reveals the existence of the benzenoid ring of DBSA in the polymer matrix in Figure 3b [28]. Sulfonate anions, –SO_3_^−^, have a stretching vibration of S=O at 1170 cm^−1^, which compensates for the cation in the polypyrrole chains. The DBSA displays the distinctive signal at 652 cm^−1^ in the PPy sample [29]. The stretching vibration of C=C can be seen at 1548 cm^−1^, whereas the stretching vibration of C–N in the Py ring can be seen at 1454–1471 cm^−1^. The signal at 1703 cm^−1^ is due to the out-of-plane wagging of the carbonyl group. At 1035 cm^−1^, the stretching vibration of C–H of the Py ring can be noticed [30,31]. The peak at 1291 cm^−1^ is connected to C–N in a plane.

All of the typical peaks of PPy are seen in the FTIR spectra of PPy/GA composites, as explained above and shown in Figure 3b. The stretching vibration of the O–H bond is responsible for the wide and low-intensity peak at 3209 cm^−1^. The stretching vibration of the C=O bond of the carboxylate group of the GA molecule is responsible for the high peak intensity at 1683 cm^−1^ [32,33]. The asymmetric stretching causes the strongest band at 1602 cm^−1^, whereas the symmetric stretching vibration of the carboxylic acid salt –COO^−^ [34] causes the weaker band at 1422 cm^−1^. Some of the GA peaks are superimposed over the PPy in the composites, indicating that the GA particles have been effectively incorporated into the PPy matrix.

### 3.3. SEM Analysis of PPy and PPy/GA Composites

**Figure 4:**
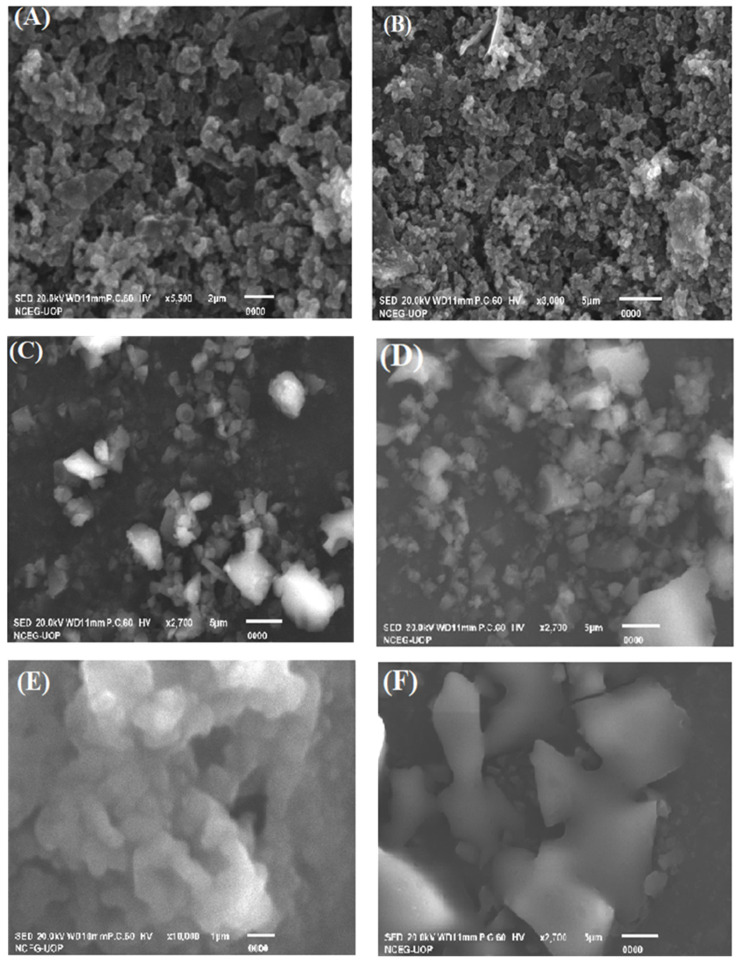
SEM images of (**A**) PPy, (**B**) PPy/GA 1, (**C**) PPy/GA 2, (**D**) PPy/GA 3, (**E**) PPy/GA 4, and (**F**) PPy/GA 5.

Figure 4 shows SEM images of pure PPy and PPy/GA 1–5 composites. As can be seen, the synthesized PPy appears to have a uniform granular structure that is agglomerated and homogeneous in shape and size [35]. The pure PPy powder has an average grain size of ~0.72 μm. Pure PPy has a weakly porous morphology with a non-uniform pore size.

The morphology of PPy/GA 1 composite is radically different. The particles are irregular in size and shape at lower concentrations (0.125 percent) of GA in composites, as seen in Figure 4B. By raising the GA content to 0.75 percent, wool morphologies with elongated and linked particles were found in the micrometric range. The particles aggregate and form large-sized particles with no discernible morphology when the GA concentration is raised even further to 1% [36]. As a result, the presence of GA has a significant effect on the size and morphology of the resulting composite materials. The overall morphology of the composites appears to be more compact and mesoporous as compared to the pure PPy. Figure 4F shows that the surface of the synthesized nanocomposite is smoother than that of PPy, which is most likely due to the hydration behavior effect of GA on PPy structure. As a result, their intrinsic viscosity and particles size are affected [36,37].

### 3.4. X-ray Diffraction

**Figure 5:**
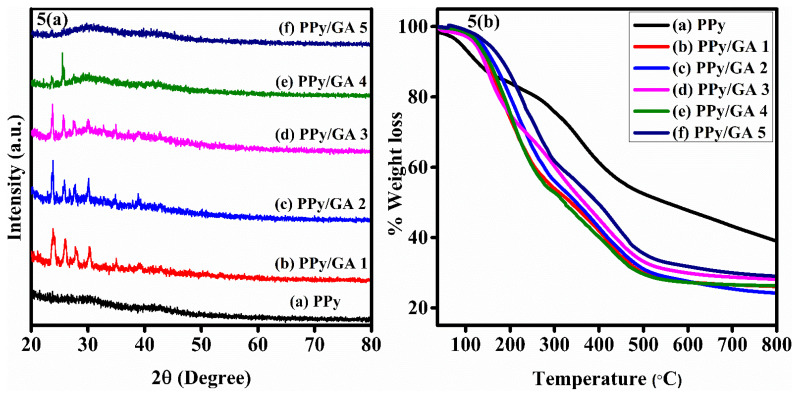
(**a**) XRD peaks of neat polypyrrole and its composites with gum arabic. (**b**) TGA of neat polypyrrole and its composites with gum arabic.

The most efficient method for analyzing the structure and nature of materials is XRD. The XRD spectrum of PPy is shown in Figure 5a. A broad peak can be seen at 2θ = 29.73°. This is PPy characteristic peak. The scattering of X-rays from PPy chains at interplaner spacing causes the peak to expand [36]. Broad peaks in the CPs are normally thought to suggest a semicrystalline structure. The PPy average chain separation from the maxima may be calculated using Equation (1) below [38].

(1) $$S=\frac{5\lambda }{8sin\theta }$$

where *S* denotes the polymer chain separation, *λ* is the wavelength of the X-ray that was used, and *θ* is the angle of diffraction at the amorphous halo’s maximum intensity. The average separation of the polymer chains was found to be 1.4 Å in the case of PPy. The Debye–Scherrer Equation (2) [39] was used to calculate the average crystallite size of PPy.

(2) $$D=\frac{k\lambda }{\beta cos\theta }$$

*D* stands for the average crystallite size, while *k* stands for the dimensionless shape factor, which has a value of 0.9, which is close to unity. It varies depending upon the crystallite’s shape, *λ* is the wavelength of X-ray that was used, and *β* is the angle of diffraction at full width and at half maximum. PPy has a crystallite size of 0.627 nm. Peaks were found at 2θ = 23.82°, 25.97°, 27.57°, 30.41°, and 34.99° in the PPy/GA 1 composite as shown in Figure 5a. The PPy/GA composites are partially amorphous and also partially crystalline in nature. The sharp and narrow peaks show the crystalline nature of the composites, whereas the halo and broad peaks show the amorphous nature of the composites. According to the previous discussion, increasing the quantity of GA in the composites to PPy/GA 2, where the amount of GA is 0.25 percent, enhances the crystallinity of PPy [40]. By further increasing the amount of GA in the composite materials, the composites become less crystalline at PPy/GA 5. GA is mostly amorphous in nature. The crystallinity of PPy/GA 1 increases due to the formation of composites of GA with PPy, which is not an unexpected result. However, at higher concentration of GA in the composites (PPy/GA 5) the amorphous nature becomes dominant as clearly seen in Figure 5a, which is attributed to the mostly amorphous nature of GA.

### 3.5. Thermal Gravimetric Analysis (TGA)

The thermal stability of pure PPy and PPy/GA composites was investigated by thermal gravimetric analysis (TGA). The TGA curves of pure PPy and PPy/GA composites are shown in Figure 5b. The thermograms were recorded in the nitrogen atmosphere by heating the samples at a temperature ranging from 25 to 800 °C. The TGA of PPy shows weight loss at three stages. The first stage of weight loss (10%) from 35 to 150 °C is attributed to the volatilization of water molecules from the polymer. The second stage of weight loss, from 150 to 400 °C, is attributed to the decomposition of the dopant molecule DBSA. The PPy backbone is completely decomposed in the third stage of weight loss from 440 to 800 °C [41].

All the composites show the same thermogram and also show weight loss at three stages. Water molecules are removed during the first stage of weight loss, which occurs at low temperatures ranging from 35 to 150 °C. At temperatures ranging from 300 to 480 °C, the degradation of GA components causes the second readily apparent weight loss. The third and final weight loss occurred at a high temperature of 500 °C, which is attributed to the decomposition of the PPy backbone. During the polysaccharide degradation process, there is no significant difference, and at high temperatures, approximately the same amount of residue is obtained [42]. The degradation of the PPy chain has been pushed to 500 °C in the composite form. This indicates that the GA has been integrated into the PPy matrix, and it also improves the composites’ thermal stability at 500 °C. The degradation of the pure PPy chain begins at 450 °C, whereas the degradation of the main polymer chain in composites begins at around 500 °C. This implies that the PPy/GA composite has good thermal stability. At 800 °C, the overall stability of PPy is greater than that of PPy/GA composites. Pure PPy causes less overall weight loss than the PPy/GA composite.

### 3.6. Electrochemical Properties

#### 3.6.1. Cyclic Voltammetry

**Figure 6:**
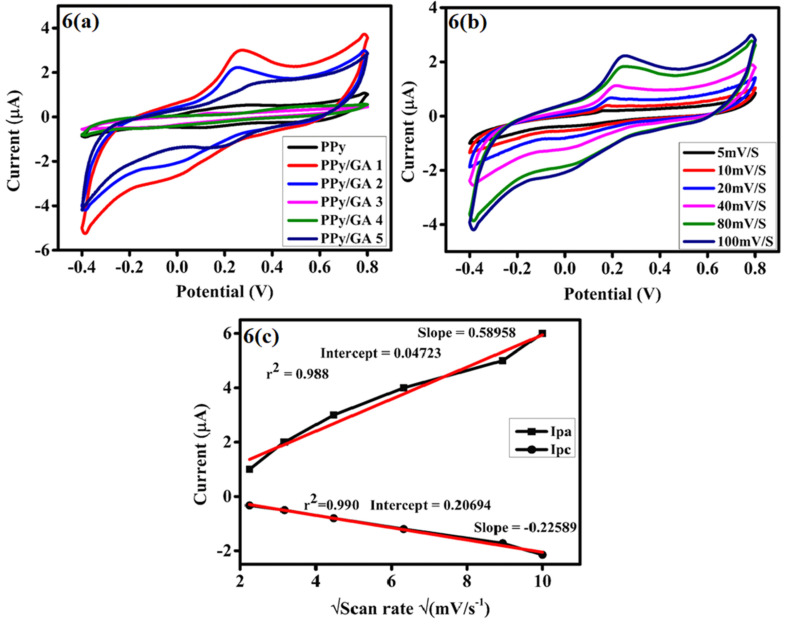
(**a**) CV of neat polypyrrole and its composites with gum arabic. (**b**) CV of PPy/GA 1 on gold electrode in 1 M H_2_SO_4_ at different scan rates. (**c**) Plot of square root of scan rates versus current for PPy/GA 1 in 1 M H_2_SO_4_.

Figure 6a shows the CV curves of PPy and PPy/GA 1 to PPy/GA 5 at the scan rate of 100 mV/s in the potential window of −0.4 to 0.8 V. PPy shows the oxidation peak at 0.31 V with a current 0.539 μA and the reduction peak at 0.107 V with −0.481 μA current. However, the PPy/GA 1 shows the oxidation peak at 0.266 V and 2.99 μA, and the reduction peak at 0.014 V and −2.59 μA. Meanwhile, the PPy/GA 2 shows the oxidation peak at 0.24 V and 2.21 μA, and the reduction peak at 0.017 V and −2.12 μA. Similarly, the PPy/GA 3/4/5 show the oxidation peak at 0.13 V/0.52 V/0.35 V with current 0.164 μA/0.47 μA/1.55 μA and the reduction peak at 0.11 V/0.12 V/0.16 V with current −0.48 μA/−0.51 μA/−1.3 μA, respectively.

Figure 6a shows that pure PPy, PPy/GA 2, and PPy/GA 5 composites have a rectangular shape, indicating that the material has a high capacitance characteristic. The shape of the PPy/GA 3 and PPy/GA 4 is similar to that of a banana. In comparison to pure PPy, the PPy/GA 1, PPy/GA 2, and PPy/GA 5 composites demonstrate high current. However, the PPy/GA 3 and PPy/GA 4 display low current as compared to pure PPy. The increase in current in case of PPy/GA 1 is due to the addition of 0.125% GA. The current reduces when further content, i.e., 0.25% of GA is added to the PPy, as seen in Figure 6a in sample PPy/GA 2. This is due to the blockage of the active site(s) of PPy. In addition, the inclusion of 0.5% and 0.75% GA, as in PPy/GA 3 and PPy/GA 4, respectively, resulted in higher active sites’ blocking. The addition of 1% GA (PPy/GA 5) causes an increase in current, which is attributable to the alcoholic group and carboxylate ion of the GA [37].

#### 3.6.2. Effect of Scan Rate on CV Curves of PPy/GA 1 Composite

To investigate the influence of scan rate, i.e., 5 to 100 mV/s, on the PPy/GA 1 composite or charge storage mechanism of the fabricated electrode can be determined by power law, where current is directly proportional to scan rate. Ip = a v^b^, where a and b are adjustable parameters, Ip is current densities and v is scan rate. The b-value is calculated from slope of straight line equation. There are two kinds of behavior battery type (b = 0.5) and capacitive types (b = 1) [43]. When the scan rate was raised, the anodic and cathodic peak currents rose, and the Ipa peaks moved slightly to the right as shown in Figure 6b,c shows a linear relationship between square root of scan rate and anodic and cathodic peaks current. Form straight line equation both Ipa and Ipc, the linear plots of current versus square root of scan rate exhibit regression values of 0.988 and 0.990 and slope values (0.5 Ipa) and (−0.2 Ipc) as shown in Figure 6c. Therefore, the reaction mechanism is suggesting that the reaction is diffusion controlled.

#### 3.6.3. EIS Study of PPy and PPY/GA Composites

**Figure 7:**
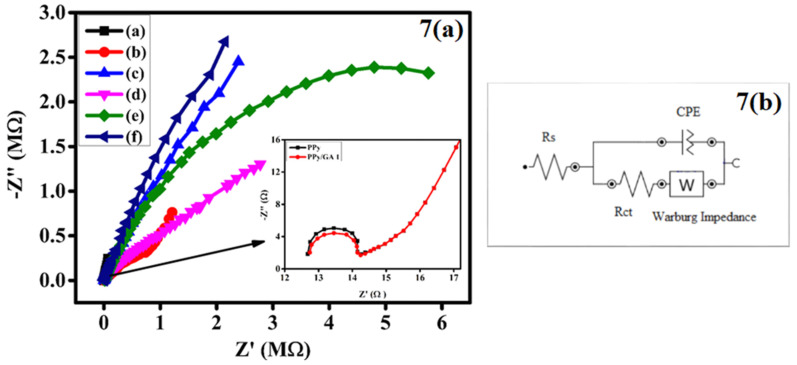
(**a**) EIS spectra of (a) PPy, (b) PPy/GA 1, (c) PPy/GA 2, (d) PPy/GA 3, (e) PPy/GA 4, and (f) PPy/GA 5. (**b**) Equivalent circuit for EIS.

Impedance spectroscopy is very beneficial for obtaining information about the electrode materials’ resistive and capacitance properties. At a constant DC potential of 0.5 V with an AC of 0.01 V, a potentiostatic EIS study was performed from 0.1 Hz to 100 kHz. Figure 7a depicts a Nyquist plot of pure PPy and various PPy/GA composites. However, Figure 7b shows an equivalent circuit for EIS. The Nyquist plot of pure PPy reveals a distorted semicircle at a high-frequency region [44]. This semicircle is followed by a 45° slanted or sloped line, which is followed by a straight line in the low-frequency region. The intercepts on the *X*-axis and the real axis are termed solution resistance (Rs), and the diameters of semicircles indicate electrode resistance (Rct) in the high-frequency area because of charge transfer resistance in the active compounds. When compared to pure PPy, the PPy/GA 1 composite had a somewhat narrower semicircle, indicating a low Rct value. In the low-frequency region, the PPy/GA 2, PPy/GA 3, and PPy/GA 5 display a straight line with an angle of 45° to 65°, which resembles an ideal capacitor and fast ion diffusion in electrode materials. In the high-frequency region shown in Figure 7a, as the GA loading in the composite increases from 0.125 to 1 wt%, Rs increases from 12.8 to 2682 ohm, and the diameter of the semicircle (Rct) grows. Despite the composites’ 0.5 wt% GA content, the high Rct suggests that as GA content increases, the number of surface electrochemical reaction sites decreases. When the GA loading in the composite is increased to 1 wt%, the number of bulk electrochemical reaction sites increases relative to the number of surface electrochemical reaction sites and the Rs again decrease. Because of the non-homogeneity of samples, porosity, and non-uniform distribution of current, a constant phase element (CPE) is used in the equivalent circuit instead of a capacitor. PPy/GA 4 indicates poor contact between the current collector and active materials, as well as high intrinsic resistance of the active material. The high resistance to ion transport between the electrolyte solution and the electrode interface causes the semi-circle or Rct value to rise. The data is summarized in Table 2.

#### 3.6.4. Galvanostatic Charge-Discharge (GCD) Study of PPy and PPY/GA Composites

**Figure 8:**
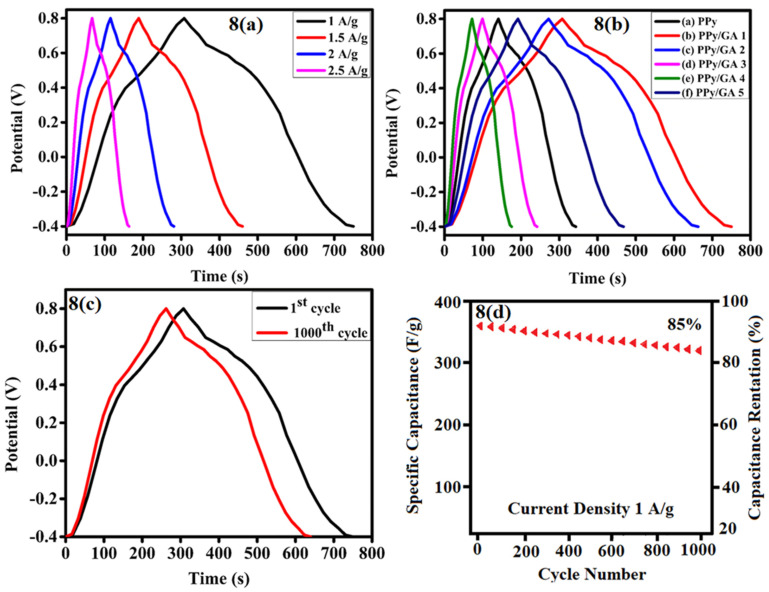
(**a**) GCD curves of PPy/GA 1 composite at different current densities. (**b**) GCD curves of neat PPy and PPy/GA composites at 1 A/g current density. (**c**) GCD curves of PPy/GA 1 for 1st and 1000th cycles at 1 A/g current density. (**d**) Plot of specific capacitance and capacitance retention versus cycle number.

GCD has also described the electrochemical performance of the produced electrodes [45] as well as the galvanostatic charge-discharge curves of PPy/GA 1 for the supercapacitors device at varied current densities of 1, 1.5, 2, and 2.5 A/g Figure 8a. The GCD curves for the fabricated electrodes PPy and PPy/GA 1 to PPy/GA 5 at various loading concentrations of gum arabic and at a fixed current density of 1 A/g are shown in Figure 8b. The shape of the curves depicts optimal capacitor behavior for supercapacitors. The charge curves are symmetric to discharge curves between potential intervals indicating feasibility of PPy/GA surface for supercapacitor [46].

The following equations were used to determine various parameters such as specific capacitance (Cs), energy density (E), and power density (P) from the GCD curves of modified supercapacitor electrodes [46].

The specific capacitance (Cs) of the modified supercapacitor electrodes was calculated by using Equation (3) [47].

(3) $$Cs=\frac{I\times \Delta t}{m\times \Delta V}$$

where “*I*” is the charge-discharge current (A), Δ*t* is the discharge time, “*m*” is the mass deposited on the electrode, and Δ*V* is the voltage difference in the discharge segment. The total energy density *E* (Wh kg^−1^) and power density *P* (Wkg^−1^) of the supercapacitor device were calculated using Equations (4) and (5) [48].

(4) $$E=\frac{1}{2\times 3.6}\times Cs\times \Delta V^{2}$$

(5) $$P=\frac{E}{\Delta t}\times 3600$$

In Equations (4) and (5) of energy density (*E*, Wh/kg) and power density (*P*, W/kg), *Cs* is the specific capacitance, Δ*V* is the potential window, and Δ*t* is the discharge time as mentioned previously. The values of *Cs*, *E*, and *P* are tabulated for PPy and PPy/GA composites in Table 3.

Table 4 compares the specific capacitance of PPy/biodegradable polymers-based electrodes to that of a PPy/GA composite developed in this study. Table 4 demonstrates that PPy/GA has a relatively high specific capacitance.

The apparent behavior of the GCD curves is well-adapted to the typical behavior of supercapacitors, which reveals that specific capacities have a declining nature and an increase in current density. The PPy/GA-based electrode proved its characteristic double-layer capacitance behavior as well as good electrochemical reversibility with a highly symmetric triangular-shaped charge/discharge curve [54]. The addition of 0.125% GA to the PPy matrix increases the charge and discharge time, which demonstrates the increase in the specific capacitance. The incorporation of GA in the PPy matrix may result in a mesoporous structure, which increases surface area and ionic conductivity. Figure 8c demonstrates the cyclic stability of the modified electrode, which was evaluated for 1000 charge–discharge cycles at a current density of 1 A/g and still had an 85% specific capacitance [2] Figure 8d. The ohmic drop in the GCD curves can be attributed the solution resistance.

## 4. Conclusions

In the current study, the pure polypyrrole (PPy) and its composites with gum arabic (GA) have been successfully prepared by inverse emulsion polymerization method using toluene and 2-propanol as a solvent media. UV-visible and the FTIR spectroscopy confirmed the formation of PPy/GA composites. The XRD result shows that GA has both natures crystalline and amorphous. When the concentration of GA is less it shows crystalline nature, by increasing the concentration of GA the amorphous nature become dominant. The SEM morphologies demonstrate porous morphology for pure PPy and compact and mesoporous morphology for PPy/GA 1 composites. PPy/GA composites show high thermal stability up to 800 °C. The synthesized material shows good electrochemical properties in terms of using cyclic voltammetry, galvanostatic charging–discharging, and EIS tests. PPy has the lowest specific capacitance, energy density, and power density, with values of 168.6 F/g, 33.698 Wh/kg, and 599.37 W/kg respectively. With a 0.125 wt% loading of gum arabic in polypyrrole, these values were enhanced to 368.57 F/g, 73.667 Wh/kg, and 599.609 W/kg, at a current density of 1 A/g.

## Figures and Tables

**Table 1:** The designation and composition of the PPy/GA composites.

Sample Designation	DBSA (mL)	BPO (g)	GA (wt%)
PPy/GA 1	0.5	0.303	0.125
PPy/GA 2	0.5	0.303	0.25
PPy/GA 3	0.5	0.303	0.5
PPy/GA 4	0.5	0.303	0.75
PPy/GA 5	0.5	0.303	1

**Table 2:** Solution resistance of the PPy/GA 1–5 composites.

S. No	Samples	Solution Resistance (Rs) (Ω)
1	PPy/GA 1	12.75
2	PPy/GA 2	279
3	PPy/GA 3	2682
4	PPy/GA 4	1456
5	PPy/GA 5	98

**Table 3:** The specific capacitance, energy density, and power density of PPy and PPy/GA 1–5.

Sample	Current Density (A/g)	Specific Capacitance (F/g)	Energy Density (Wh/kg)	Power Density(W/kg)
PPy	1	168.6	33.698	599.37
PPy/GA 1	1	368.57	73.667	599.609
PPy/GA 2	1	325.83	65.12	599.607
PPy/GA 3	1	118.83	23.75	599.579
PPy/GA 4	1	86.25	17.238	599.582
PPy/GA 5	1	230	45.97	599.608

**Table 4:** Comparison of specific capacitance of PPy/biodegradable polymers-based electrodes.

Electrode Material	Specific Capacitance	Current Density	Reference
PPy/lignin	11.42	2.0A g^−1^	[49]
PPy/Nanocellulose	35 F g^−1^	0.27 A g^−1^	[50]
PPy/Cotton	225 F g^−1^	0.6 mA cm^−2^	[51]
PPy/BC	316 F g^−1^	0.2 A g^−1^	[52]
PPy/SFC	367 F g^−1^	0.2 A g^−1^	[53]
PPy/GA	368.57 F g^−1^	1.0 A g^−1^	This work

## Data Availability

Not applicable.
